# A Foot-Arch Parameter Measurement System Using a RGB-D Camera

**DOI:** 10.3390/s17081796

**Published:** 2017-08-04

**Authors:** Sungkuk Chun, Sejin Kong, Kyung-Ryoul Mun, Jinwook Kim

**Affiliations:** 1 Spatial Optical Information Research Center, Korea Photonics Technology Institute, Gwangju 61007, Korea; k612051@kopti.re.kr; 2 R&D Division, LS Networks, Seoul 04386, Korea; sjkong@lsnetworks.com; 3 Center for Imaging Media Research, Korea Institute of Science and Technology, Seoul 02792, Korea; krmoon02@gmail.com

**Keywords:** biomedical image processing, RGB-D camera, foot-arch, arch width, arch height, arch index, computer aided analysis

## Abstract

The conventional method of measuring foot-arch parameters is highly dependent on the measurer’s skill level, so accurate measurements are difficult to obtain. To solve this problem, we propose an autonomous geometric foot-arch analysis platform that is capable of capturing the sole of the foot and yields three foot-arch parameters: arch index (AI), arch width (AW) and arch height (AH). The proposed system captures 3D geometric and color data on the plantar surface of the foot in a static standing pose using a commercial RGB-D camera. It detects the region of the foot surface in contact with the footplate by applying the clustering and Markov random field (MRF)-based image segmentation methods. The system computes the foot-arch parameters by analyzing the 2/3D shape of the contact region. Validation experiments were carried out to assess the accuracy and repeatability of the system. The average errors for AI, AW, and AH estimation on 99 data collected from 11 subjects during 3 days were −0.17%, 0.95 mm, and 0.52 mm, respectively. Reliability and statistical analysis on the estimated foot-arch parameters, the robustness to the change of weights used in the MRF, the processing time were also performed to show the feasibility of the system.

## 1. Introduction

The foot-arch, which plays a key role in supporting the weight of the body and providing propulsion force during push-off, is important because it enables a more natural and aesthetic gait and protects the foot from injury. It is well-known that filling the void space between the foot-arch and shoe reduces the plantar pressure, alleviates impact force, and improves shoe comfort [1,2]. Therefore, understanding the geometric shape of an individual’s foot and foot-arch is necessary to provide direct and useful information, not only for clinical and rehabilitative purposes, but also for designing personalized and comfortable footwear [3].

The arch index (AI), the arch width (AW), and the arch height (AH) are the representative parameters showing the foot characteristics of healthy individuals as well as subjects with foot functional abnormalities. These parameters are defined based on the shape of the footprint. The AI is defined by the ratio of the midfoot area to the entire foot area (excluding the toes) from the measured footprint [4]. The AW and the AH are defined as the vertical and horizontal distances from the midpoint of the medial border line (MBL), which is the line connecting the most medial border of the metatarsal and heel region of the foot [5], in the arch region of the footprint to the foot surface. Based on these definitions, the traditional methods measure these parameters manually using a footprint on a grid paper. Therefore, the accuracy of the measurement is dependent on the measurement skill of the experimenter, and reliability and repeatability are thus usually poor.

In order to overcome this problem, many scientific researchers have attempted to measure and analyze the foot shape via vision-based measurement (VBM) approaches. VBM involves producing measurement results using 2D/3D visual images and computational methods. The main idea of this approach in the context of biometric measurement of geometric shape of human body parts is that a camera or a scanner captures the human body surface and measurement is done by analyzing the captured visual and geometric data using hardware and software technologies [6]. Recent rapid advances in imaging devices and computing systems have allowed VBM to be easily used to measure, detect, monitor, recognize, and record physical phenomena in a variety of automated applications and scenarios: measurement of human body parts [7], human motion tracking [8], hand recognition [9], face recognition [10], gait analysis [11], palmprint-based identifications [12], and 3D human body reconstruction [13].

In this study, we aimed to develop an autonomous geometric foot-arch measurement platform that is capable of capturing the sole of the foot and estimating three foot-arch parameters: AI, AW and AH. The proposed system captures 3D geometric and color data on the plantar surface of the foot in a static standing pose using a commercial RGB-D camera installed below the transparent footplate. As explained in [4,14,15], three foot-arch parameters can be calculated based on the foot axis and the MBL in the footprint image representing the contact region of foot. Therefore, in this paper, we describe the process of detecting the contact region of foot and computing the foot-arch parameters using the 3D geometric and color data obtained from the RGB-D camera and validate the results of the estimated foot-arch parameters from human experiment. The main contributions of this work are:

- *A new methodology for sole of foot analysis*: The proposed system, which automatically analyzes the plantar surface of the foot in a static standing pose, utilizes a commercial RGB-D camera installed below the transparent acrylic plate of the scanning stage. Some existing methods using separately designed wearable devices or visual markers involved adding an extra factor to the foot surface, but this is undesirable in a clinical environment. Also, some use camera-projector systems or multiple cameras to reconstruct the surface of the foot. However, these are more expensive than commercial off-the-shelf RGB-D cameras and require heavy computational processing. In contrast, our system can measure the plantar surface of the foot efficiently by using an RGB-D camera, which provides accurate 3D geometric and visual information.
- *An automatic foot-arch parameter computation method*: To define and recognize numerically the characteristics of individual feet, the system automatically calculates the foot-arch parameters—AI, AW, and AH. The system detects the contact region from an input color and depth image set by applying image segmentation methods such as data clustering and Markov Random Field (MRF) techniques, and generates the foot-arch parameters by analyzing the 2D and 3D shape of the contact region. In contrast to other existing systems that focus mainly on surface reconstruction and footprint image generation, our system is capable of not only capturing the sole of the foot but also determining the three foot-arch parameters.

## 2. Related Literature

VBM-based foot shape analysis systems can be classified into passive and active 3D shape measurement techniques, depending upon the sensing method used [16]. The first is based on matching of corresponding pixels in multiple images captured by multiple cameras. The second involves measurement of the 3D shape by emitting and receiving light. As one of the earliest passive 3D shape measurement systems, Lee et al. proposed a foot model reconstruction using 12 cameras [17]. From the 12 captured images, the system calculates major foot parameters, such as foot length and ball width, to scale the foot model in their database. Reconstruction is completed by morphing and deforming the foot models in the database similar to the user’s foot. Coudert et al. proposed a six cameras-based method of measuring 3D foot deformation during the gait cycle [18]. The final foot model is reconstructed by image matching and triangulation techniques. Amstutz et al. used ten cameras to reconstruct the 3D foot shape [19]. They fitted the initial 3D model to the real foot by projecting the initial model to the 2D images of the real foot. In [20], Al-Baghdadi et al. applied a dense surface modeling algorithm to automatically obtain a 3D point cloud of a human foot. A mounting platform for three video cameras and a glass-top step-on platform were used to capture the foot. Alshadli et al. introduced a video based 3D foot modeling system to map the shape changes of the medial longitudinal arch during gait [21]. The imaging system they proposed consists of 4 high definition video camcorders and a force plate. Using this system, multiple images and force data synchronized with the camcorders can be captured simultaneously. The 3D foot shape is reconstructed by using multiple images. In [22], Alshadli proposed the method to calculate the foot mobility magnitude (FMM) and arch height index (AHI) using the system proposed in [21]. And the author tried to find the relationship between the dynamic FMM and AHI and the foot posture index (FPI). These camera-based reconstruction methods have limitations in that a texture of the foot surface for correspondence matching between multiple images is required for an accurate foot shape acquisition. To overcome these limitations, they proposed special socks or painting of the texture on the foot skin. Although these solutions were suitable for their reconstruction process, they are undesirable in a clinical environment and not comfortable.

The active shape measurement methods typically acquire 3D shape by the structured light method or time-of-flight (ToF) method. The structured light method calculates the distance by using the shape and location of the projected pattern. The ToF method calculates the distance by measuring the time-of-flight of a light signal to the subject. These methods are widely used, as users do not need to wear or attach sensors or markers to their feet. In [23,24], they introduced a camera-projector system to reconstruct the plantar surface. A pattern comprising small squares of random colors is projected onto the foot sole and the reflected patterns are captured by the camera; these are used to reconstruct the 3D geometric shape of the sole. JezerĹĄek et al. presented a multiple laser plane triangulation technique for high-speed foot measurement [16]. They used four measuring modules, each of which comprised a laser projector and a digital camera. In [25], Novak et al. developed a 3D foot scanning system with a rotational laser-based measuring head. In their system, the measuring head, comprising a three laser line projection unit and two cameras, rotates around the center of the platform on which the customer stands, and measures both feet simultaneously. Herrewegen et al. used four structured light scanning modules to capture the foot shape [26]. To analyze multi-segmental foot kinematics, the proposed system tracks four segments (shank, calcaneus, metatarsus, and hallux) during walking using the iterative closest point (ICP). Using the ToF camera-based foot shape measurement system proposed in [27], Samson et al. proposed a new method of analyzing foot roll-over under dynamic conditions [28]. The system generates sequential images of lowest height data (LHD), which represent the distance from the 3D foot shape to the ground plane by projecting the foot surface. For each frame during foot roll-over motion, the change in mean height and projected surface in seven regions of interest (ROI) are computed. In [29], Chen et al. proposed a hand held RGB-D camera based 3D foot parameter estimation method. The user firstly rotates around the foot and captures the consecutive color and depth images using a hand held RGB-D camera. The system reconstructs the 3D shape of the foot with reference to the AR code located around the feet. And then using the 3D shape of foot, the system calculates the foot length, width, and ball girth. Even though their system uses 3D shape information and provides useful information for suitable shoe selection, they do not provide any essential information to calculate AI, AW, and AW such as the contact region of foot. Although some existing systems can measure the entire surface of the foot and some provide analytic information related to foot measurement, such as the deformation of cross-sections [23], global foot dimensions (foot length, width, height, and girth) [25,29], and changes in mean height and projected surface [28], the studies do not include any information regarding the AI, AW, and AH which can be used for clinical purposes and design of ergonomic personalized footwear.

In this paper, among many features of the foot, we focus on automatic estimation of three foot-arch parameters, AI, AW, and AH. The AI is measured by counting the squares in the graph paper with which the sole of foot is in contact. The AW and AH are measured manually using a ruler. These conventional methods are highly dependent on the measurer’s skill and undesirable in a clinical aspect since the subject must cover his/her foot in ink to create the footprint image. In [30], Chu et al. proposed an image processing-based system to improve the accuracy and repeatability of the footprint acquisition and AI calculation of the traditional method. However, it is impossible to compute and predict the AI, AW, and AH simultaneously using the footprint-based method. This is because the footprint does not contain 3D information of the foot, such as the height of the plantar surface—it represents simply the 2D contact region of the sole of the foot.

The system proposed in this paper is a commercial RGB-D camera-based foot-arch measurement system for AI, AH, and AW computation. Compared with the passive 3D measurement methods, the system is easily able to obtain 3D shape of sole of foot from the geometric and visual data captured by the RGB-D camera, without special socks or painting the pattern on the foot skin. Another advantage that the proposed system includes that the used camera is compact, relative low cost, and the fast data acquisition frame rate. The frame rate and image resolution of the system in [28] are 40 Hz and 176 × 144. The frame rate and image resolution of the used camera in the proposed system are 60 Hz and 640 × 480. Even though the system in [23] has higher image resolution (1024 × 768) than the proposed system, the frame rate is slow (14 Hz). In [29], they use the low-cost RGB-D camera similar with the RGB-D camera used in the proposed system. However, the frame rate and image resolution are relatively low as 30 Hz and 320 × 240, and also their system cannot capture the plantar shape of the foot. In addition, unlike existing VBM studies that analyze the overall shape change of the foot, the proposed system calculates AI, AW, and AH that reflect the characteristics of the foot-arch. Therefore, for the foot-arch parameters estimation, the proposed system has advantages of better usability and convenience than the existing systems.

In particular, the conventional footprint-based foot-arch parameter measurement method depends on manual operation, but the proposed system efficiently computes them through an image segmentation technique and computational 3D shape analysis of foot. Moreover, since the proposed system is able to estimate the AI, AW, and AH simultaneously using the advantages of the 3D shape information of foot, it is more convenient to measure than the conventional method. And also another advantage of the proposed system compared with the 2D footprint based method is the calculation time. The conventional method takes an average of 3 min or more, from painting ink on the foot to calculating the AI based on the inked area of the footprint and measuring the AH using a ruler or a caliper. The 2D digital image processing based method proposed in [30] takes 10 s or more to calculate AI. However, the proposed system is able to calculate not only AI but also AW and AH simultaneously within 10 s (Section 5.5). The other advantage is that the proposed method can be applied to the dynamic foot motion analysis. The camera used in the proposed system can capture shape and color data for continuous foot motion. This can be used to analyze how the user's feet change in the stance phase of the gait. For example, successive 3D shape and color information of foot can be obtained to analyze how foot shape changes at each stage of the stance phase, which consists of heel strike, foot flat, midstance, heel off, and toe off. However, 2D footprint based technologies cannot be employed for dynamic foot analysis, since the acquired data does not reflect the 3D dynamic foot shape change. In this system, we focus on the static foot measurement, and do not dynamic foot analysis.

## 3. Foot-Arch Parameters

To identify the important considerations in system development, foot-arch parameters must be examined. In this section, we briefly describe the considerations of our system with respect to the computational method for estimation of foot-arch parameters.

**Figure 1:**
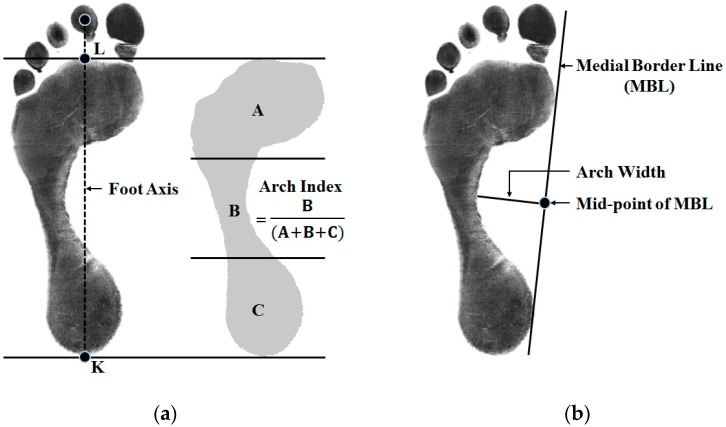
Definitions of foot-arch parameters: (**a**) arch index (AI) measurement from the footprint, (**b**) arch width (AW) and height (AH) measurement.

The AI developed by Cavanagh and Rogers represents the ratio of the area of the middle third of a footprint relative to the total area (excluding the toes) [4]. The definition of AI is as follows: The line of the foot axis between the centers of the heel (point K in Figure 1a) and the second toe is measured. Next, a perpendicular line is drawn tangential to the most anterior point of the main body of the footprint. The intersection point is then marked (point L in Figure 1a). The line L-K is divided into three equal parts. The main body of the footprint is divided into three areas by those points with the perpendiculars from the foot axis. The AI, the ratio of the middle area relative to the total area, is computed [14].

The AW and AH are clinically important, as they are closely related to foot type. Low arches or flat feet can cause heel pain, arch pain and plantar fasciitis. High arches can cause plantar fasciitis as the plantar fascia is stretched away from the calcaneus or heel bone. The AW and AH are generally measured using the footprint and a ruler, as follows. The MBL is first drawn. Then, a perpendicular line is drawn from the mid-point of the MBL in the arch area to the mid-foot. The length of this line is the AW [15]. The AH is defined as the length of a perpendicular line from the mid-point of the MBL to the plantar surface of the foot. Figure 1b shows the lines and points required to measure AW and AH.

To calculate the AI, AW, and AH, the region of the foot in contact with the floor, which can be easily measured using the footprint, must be defined. Also, the key points and lines, such as the center of the heel and the second toe and the MBL, are required. Therefore, from the next section, we describe how the proposed system calculates the foot-arch parameters by solving the following two technical problems: how to recognize the region in contact with the footplate using the color and depth image set and how to define the key points and lines.

## 4. Method

**Figure 2:**
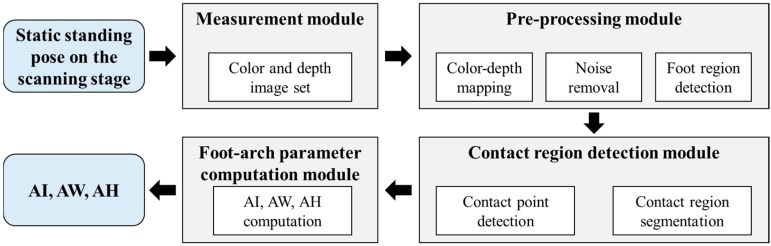
System flow of the proposed system.

The system consists of measurement and analysis modules. The measurement module includes the scanning stage and a RGB-D camera underneath transparent acrylic board mounted on the scanning stage (Section 4.1). And the analysis module is programmed to process the obtained color and geometric data of foot and to extract the foot-arch parameters through the following three submodules: pre-processing (Section 4.2), contact region detection (Section 4.3), and foot-arch parameter computation (Section 4.4). The system flow is shown in Figure 2.

### 4.1. Measurement Module

**Figure 3:**
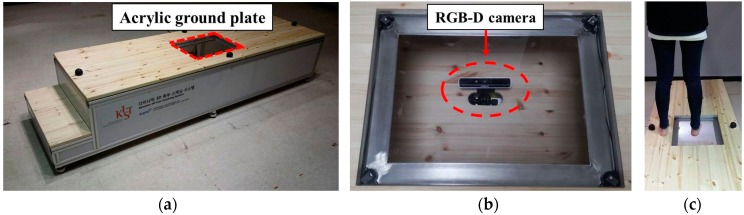
System installation: (**a**) scanning stage of the proposed system, (**b**) RGB-D camera beneath the transparent acrylic board, (**c**) sole of foot measurement using our system.

The scanning stage used to capture the plantar surface of the foot is 45 cm in height and 70 cm in width. A transparent acrylic board of 45 cm × 35 cm is embedded at the middle of scanning stage. This acrylic board is used as the footplate where the targeted foot is measured on. For stable lighting condition, a led desk lamp is installed inside of the scanning stage. A RealSense F200 RGB-D camera (Intel, Santa Clara, CA, USA) is installed 30 cm beneath the footplate to measure the sole of the foot (Figure 3).

The camera captures and sends the input data in the form of a color and depth image set containing visual and geometric information on the plantar surface of the foot to a server via a USB cable. Using the data obtained, the system analyzes the foot shape in terms of the contact region, arch index, width, and height. These outputs can be transferred to other foot analysis systems.

### 4.2. Pre-Processing Module

The preprocessing module (PM) performs noise removal measured from an input depth image and maps the input color image to the depth image. Each pixel in an input depth image represents the distance from the camera to the surface along the optical axis of the camera, and can easily be converted into 3D points (x,y,z). However, direct use of the depth image are not recommended due to optical noise, lost depth information on shiny surfaces, and flickering artifacts. To increase the quality and stability of the depth image, the PM reduces the noise in the input depth image using median filtering to preserve foot edges while removing noise [31]. In the proposed system, a 3×3 sliding window is used, and the median depth value among nine pixels in the window is taken as the filtered depth value.

To use visual and geometric information simultaneously, their coordinate systems must be unified since the color and the depth images captured from the RGB-D camera have different coordinates. The calibration process is necessary to find the intrinsic parameters of each camera, such as lens distortion and focal length, as well as extrinsic parameters representing relative poses between two cameras. In our system, the camera is calibrated using the method proposed in [32]. Using the calculated intrinsic and extrinsic parameters, the system maps the input color image to the depth image. As a result, a pixel in the depth image becomes a 6D point (x,y,z,r,g,b), where (x,y,z) is the 3D position of the point in the depth camera coordinate, and (r,g,b) is the color information mapped from the input color image.

**Figure 4:**
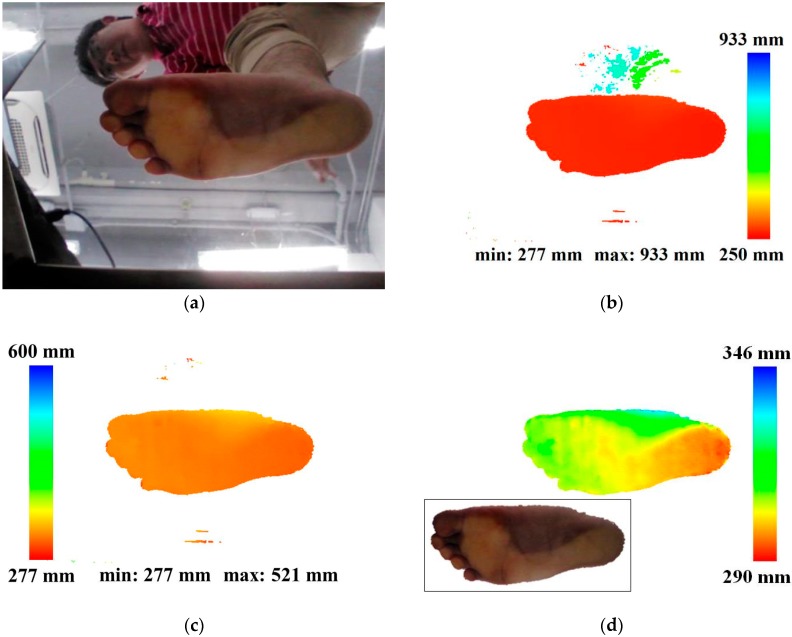
Results of preprocessing module: (**a**) input color image, (**b**) input depth image color-coded by depth value, (**c**) depth image filtered by depth thresholding, (**d**) foot point image filtered by the connected component labeling and color mapped image (left bottom).

Additionally, the system first filters out the points whose distances from the camera are more than the predefined threshold (600 mm in this system) to remove the points which does not corresponds to the foot-point (Figure 4c). then retains only the largest component by applying connected component labeling to remove other unnecessary minor noisy points [33]. Connected component labeling involves identifying all connected components in an image and assigning a unique label to all pixels in the same component. The computation of size, position, orientation and bounding rectangle can be carried out using the result labels. The system applies the labeling algorithms to detect foot points by retaining only the largest component and filtering out the others.

To find the largest component as the group of foot points, the system first converts the filtered depth image to a binary image. In the binary image, two-pass-based connected component labeling is applied. In the first pass, the system scans the binary image left to right and top to bottom. If the pixel is 1, the system assigns a label to the pixel as follows: (1) If the left pixel is 1 and the top pixel is 0, assign the label of the left pixel. (2) If the top pixel is 1 and the left pixel is 0, assign the label of the top pixel. (3) If the top and left pixels are 1, assign the label of the left or top pixel. If the two labels of the left and top pixels are different, record that the two labels are equivalent. In the second pass, the system assigns a unique label to all pixels of a component using the lowest label for each equivalent set. Using the number of pixels in each label, the system finds the largest component, and defines the corresponding pixels as the foot points. Figure 4d shows the detected foot points and depth information. Invalid points are filtered out and only the foot points are retained.

### 4.3. Contact Region Detection Module

The foot-arch parameters are computed based on the contact region. To identify the contact region, the system first detects the contact points and then defines the contact region by a Markov random fields (MRF)-based method.

#### 4.3.1. Contact Point Detection

A contact point is defined as a point whose distance from the footplate (acrylic board) is ideally zero. If the RGB-D camera is ideally installed parallel with the footplate, the distance between the foot point and the footplate could be calculated as the difference between the depth value of a point in the depth image and the distance of the footplate from the camera. However, it is almost impossible to install the camera in such a way, and the camera is generally rotated. Therefore, recognizing the rotation and translation of the footplate in 3D space is required to calculate the distance from the foot point to the footplate. One of the simplest ways to recognize the footplate is to model the plane using, for example, a checkerboard. However, this plane modeling method must be performed whenever the camera installation is changed. To overcome this problem, the proposed system automatically estimates the footplate from the previously filtered foot points based on the following assumption: among the foot points in the depth image, those in the contact region comprise the greatest proportion and their surface normal vectors are identical. Using this assumption, the system calculates the unit normal vectors of the foot points, clusters them, and finds the largest cluster. Finally, the system estimates the plane equation using the points included in the detected cluster.

The point neighborhood-based surface normal vector estimation method is used in our system [34]. This approach first computes two 3D vectors between the left and right neighboring points and between the upper and lower neighboring points in the depth image. The normal vector is computed using the cross product of the two vectors. This method is considerably faster, but sensitive to noise. To alleviate this problem, the proposed system applies a smoothing filter to the normal vectors by averaging them.

**Figure 5:**
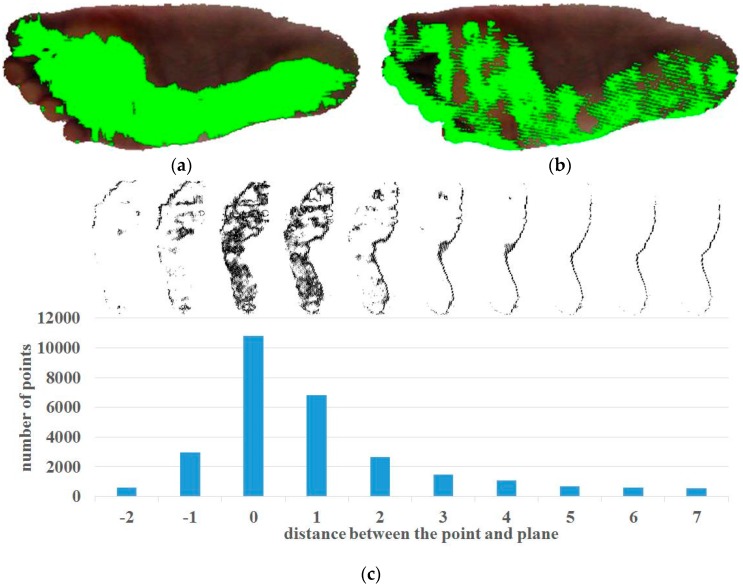
Contact point detection process: (**a**) foot points included in the largest normal vector cluster (green points), (**b**) detected contact points (green points), (**c**) number of corresponding points according to distance from the plane.

As mentioned above, we assume that most of the points are in the contact region and their surface normal vectors have similar directions. To identify the point set with similar normal vectors, the system performs clustering of the normal vectors [35]. The system constructs a 3D voxel grid and votes the normal vectors to the corresponding grid cell. In our system, each side of the 3D grid has a range of −1.0 to 1.0, and the grid consists of 20×20×20 cells. The size of each cell is 0.1×0.1×0.1. Each normal vector is voted according to its *x*-*y*-*z* coordinates. All non-empty cells are initial clusters. The system calculates the average normal vector of each initial cluster. To alleviate the discretization effects on the cell size, the system merges neighboring clusters and updates the average normal vector, if the difference between their averages is smaller than the cell size. After the merging process, the points in the largest cluster are used to compute the plane equation estimation. Figure 5a shows the results of our normal vector clustering algorithms.

The plane equation is calculated using the least-squares plane fitting algorithms proposed in [36]. A plane equation can be specified by a point ($x_{0},\;y_{0},\;z_{0}$) on the plane and the normal vector $n=\;{\left(n_{x},\;n_{y},\;n_{z}\right)}^{T}$ of the plane. Any point (x, y, z) on the plane satisfies $n_{x}\left(x-x_{0}\right)+n_{y}\left(y-y_{0}\right)+n_{z}\left(z-z_{0}\right)=0$. The best-fit plane to m given data points $\left(x_{i},\;y_{i},\;z_{i}\right)$, where m≥3, passes through the centroid $\left(\bar{x},\;\bar{y},\;\bar{z}\right)$ of the data, and this specifies a point on the plane. The first step is to find the average $\left(\bar{x},\;\bar{y},\;\bar{z}\right)$ of the points. A matrix A is formulated such that its first column is $x_{i}-\bar{x}$, second column is $y_{i}-\bar{y}$, and third column is $z_{i}-\bar{z}$. Then the matrix A is decomposed by singular value decomposition. The singular vector corresponding to the smallest singular value is chosen as the normal vector n of the plane. Finally the plane with the best fit to the given data points is specified by $\bar{x}$, $\bar{y}$, $\bar{z}$, and n: $n_{x}\left(x-\bar{x}\right)+n_{y}\left(y-\bar{y}\right)+n_{z}\left(z-\bar{z}\right)=0$.

To detect the contact points among the foot points, the system exploits the distance from the estimated footplate to the point. The distance can be easily calculated using the estimated plane equation: $d=n_{x}\left(x-\bar{x}\right)+n_{y}\left(y-\bar{y}\right)+n_{z}\left(z-\bar{z}\right)$. Ideally, the distances of all contact points would be zero, but some are not zero due to the noise of the input data and the estimation error in the plane-fitting process. For this reason, the system identifies the set of contact points based on the following assumption: among the foot points, the points in the contact region comprise the greatest proportion among the foot points. The system calculates the distance from each point first and then, finds the distance to the footplate of the largest number of the foot points. Finally, the system defines the foot points with distances less than the detected distance as the contact points. Figure 5b shows the recognized contact points and Figure 5c shows the change in the number of foot points according to distance. Algorithm 1 explains the procedures of contact point detection.

**Algorithm 1** Procedures of Contact Point DetectionInput data: foot points in the input depth image Output data: contact points Variables-i: index of the foot points in the depth image-$f_{i}$: *i*-th foot point in the depth image-$n_{i}$: normal vector of $f_{i}$-$d_{i}$: distance from the footplate to $f_{i}$-c_size: length of one side of a cell in the 3D voxel grid
Procedures:
Constructing a 3D voxel grid:create a 3D voxel grid having a range of −1.0 to 1.0 and divide the grid into cells of equal size.Computing a normal vector:for all $f_{i}$,calculate the normal vector $n_{i}$ and apply a smoothing filter.Voting normal vectors:for all $n_{i}$,assign $n_{i}$ to the cell in the gridSetting initial clusters:set the non-empty cells as the initial clusters and calculate the average normal vector in the initial cluster.Merging adjacent clusters:for all inital clusters,calculate the distance between the adjacent cluster and merge them if the distance is smaller than c_size.Estimating footplate equation:find the cluster with the largest number of normal vectors of the foot points and estimate the plane equation using the correspoindng foot points.Calculating distances of foot points:for all $f_{i}$,calculate the distance from the footplate using the plane equation.Constructing a histogram:create a hisgtorm of the distance from the foot points and the footplate.Detecting the contact points:find the bin with the highest frequency and define the points corresponding to the bin as the contact points.

#### 4.3.2. Contact Region Detection

As mentioned above, the foot-arch parameters are computed based on the contact region in the plantar surface of the foot. Therefore, the system must define the contact region before computing the foot-arch parameters. In this module, the proposed system extracts the dense and connected set of points in contact with the footplate. To solve this problem, a MRF is applied to detect the contact region [37]. A MRF enables incorporation of shape, color and distance from the footplate cues in a single unified model. Using the MRF, the system segments the foot point image into the following three classes: the contact, non-contact, and background regions. The contact and non-contact region are respectively the set of foot points in contact and not in contact with the footplate. The background region is the non-foot points in the image.

Given a color image x, set of distances d, and set of included angles between the normal vectors of the points and the normal vector of estimated footplate θ, the energy function of the proposed MRF model for the class labels c is defined as:

(1) $$E\left(c,\;\pi ,\;w,\;x,\;d,\;\theta \right)=\;\sum_{i}w_{\psi }\psi \left(c_{i},\;x_{i};\;\pi_{\psi }\right)+w_{\lambda }\lambda \left(c_{i},\;d_{i};\;\pi_{\lambda }\right)+w_{\rho }\rho \left(c_{i},\;\theta_{i};\;\pi_{\rho }\right)+\sum_{\left(i,j\right)\in \Upsilon }\phi \left(c_{i},c_{j},x_{i},x_{j}\right),$$

where Υ is the set of the edges in the four-connected grid, $\pi =\left\{\pi_{\psi },\pi_{\lambda },\pi_{\rho }\right\}$ are the model parameters, $w=\left\{w_{\psi },w_{\lambda },w_{\rho }\right\}$ are the weights for each term, and i and j are the index nodes in the grid (corresponding to positions in the image).

**Figure 6:**
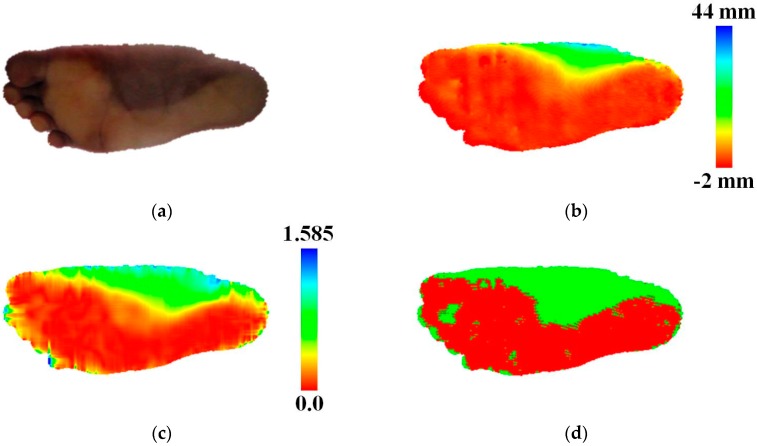
Example of MRF-based contact region detection: (**a**) color image of the foot points (**b**) visualization of distance between the foot points and the estimated footplate, (**c**) visualization of the angle between the normal vectors of the footplate and the points, (**d**) MRF-based segmentation (red: contact region, green: non-contact region, and white: background).

As shown in Figure 6a, the contact and non-contact regions in the foot point set have different colors due to skin deformation caused by the effect of body weight on the contact region. The first term $\psi \left(c_{i},\;x_{i};\;\pi_{\psi }\right)$, known as the color potential, is based on this idea and represents the color distribution of the class given the point color. This term is proportional to the likelihood of the input color $x_{i}$ given $c_{i}$, and is defined as:

(2) $$\psi \left(c_{i},\;x_{i};\;\pi_{\psi }\right)=-\log P\left(x_{i}|c_{i}\right),$$

where $P\left(x_{i}|c_{i}\right)$ is the normalized distribution given by the Gaussian mixture models (GMM) using learned parameters $\pi_{\psi }$. A RGB color model is used for the color potential.

The second term $\lambda \left(c_{i},\;d_{i};\;\pi_{\lambda }\right)$, known as the distance potential, captures the distance distribution of the class given the distance of the point. This term is based on the notion that many contact points are closer to the footplate than are the non-contact points, as shown in Figure 6b. This term is proportional to the likelihood of the input distance $d_{i}$ given $c_{i}$, and is defined as:

(3) $$\lambda \left(c_{i},\;d_{i};\;\pi_{\lambda }\right)=-\log P\left(d_{i}|c_{i}\right),$$

where $P\left(d_{i}|c_{i}\right)$ is the normalized distribution given by a single Gaussian model using the learned parameter $\pi_{\lambda }$.

The third term $\rho \left(c_{i},\;\theta_{i};\;\pi_{\rho }\right)$, known as the angle potential, captures the angle distribution of the class given the included angles between the normal vectors of the points and the normal vector of the footplate. The included angle can be calculated easily from the inner product of two normal vectors. This term is based on the idea that many contact points have normal vectors similar to the normal vector of the footplate, as shown in Figure 6c. This term is proportional to the likelihood of the input angle $\theta_{i}$ given $c_{i}$, and is defined as::

(4) $$\rho \left(c_{i},\;\theta_{i};\;\pi_{\rho }\right)=-\log P\left(\theta_{i}|c_{i}\right)\;\mathrm{and}\;\theta_{i}=\cos^{-1}\left(n_{p}\;\cdot \;n_{i}\right),$$

where $P\left(\theta_{i}|c_{i}\right)$ is the normalized distribution given by a single Gaussian model using the learned parameter $\pi_{\rho }$, and $n_{p}$ and $n_{i}$ are the normal vectors of the footplate and the point, respectively.

The last term $\phi \left(c_{i},c_{j},x_{i},x_{j}\right)$, known as the smoothness potential, is a prior distribution that discourages large differences in the labels of neighboring sites by assigning a low value to these configurations. We model this term using a contrast-sensitive Potts model, as follows:

(5) $$\phi \left(c_{i},c_{j},x_{i},x_{j}\right)=Ι\left(c_{i}\neq c_{j}\right)\exp\left(-\beta \|x_{i}-x_{j}{\|}^{2}\right),$$

where Ι(·) is an indicator function that is 1 (0) if the input argument is true (false), and β is a fixed parameter. The color difference between two neighboring points is used in this smoothness potential. In practice, this enforces spatial continuity of labels since the output of the term becomes large if the color difference is small and the corresponding labels are not identical.

Each of the potential terms is trained separately to produce a normalized model parameter. The training sample of each class is chosen from the result of contact point detection. The samples for the contact region class are randomly selected from the contact points. The samples for the non-contact region class are randomly selected from the non-contact points among the foot points. However, some points in the contact region are classified as non-contact points since the result point set of the contact point detection is sparse, as shown in Figure 5b. To prevent selection of points in the contact region as the samples for the non-contact region class, the system applies dilation to the contact points in the result image, removes them, and selects the samples for the non-contact region class from among the remaining foot points. The samples for the background region class are set to fixed values: ${\left[0,\;0,\;0\right]}^{Τ}$, 0, and $180^{{}^{\circ}}$ for the color, distance, and angle potentials, respectively. The system adds small noises to them. For the color potentials, an Expectation-Maximization (EM) algorithm is used to learn the GMM parameter $\pi_{\psi }$. We use three Gaussian models to train the color distribution of a single class. For the distance and angle potentials, we train the Gaussian models of each class using the mean and standard deviation. Compared with the contact points in Figure 5b, the recognized contact region is dense (Figure 6d). The weights, $w_{\psi }$, $w_{\lambda }$, and $w_{\rho }$, for color, distance, and angle potentials are set to 2.9, 9.3, and 1.8, respectively.

### 4.4. Foot-Arch Parameter Computation Module

The foot-arch parameters—AI, AW, and AH—can be defined by using the contact region and the key points and lines, such as centroid points of the second toe and heel and the MBL. In this section, we describe computation of foot-arch parameters by 2D and 3D shape analysis of the estimated contact region.

#### 4.4.1. Arch Index Computation

To compute the AI from the estimated contact region, the system performs following three processing: (1) foot axis definition, (2) toe removal, (3) AI computation.

The system defines the foot axis based on the two key points, the centers of the second toe and heel. Some of existing method presented the automatic foot axis detection [28,29]. Their methods compute the first principal axis of foot point distribution and define it as the foot axis. However, these methods are not suitable for finding the correct foot axis, since the extracted foot axis based on these methods is generally not the line connecting the centers of the second toe and heel. And since the foot data distribution depends on posture changes, the resulting foot axis also is sensitive to the posture changes. In order to accurately define the foot axis in the proposed system, the center of the second toe is manually selected by user input using a graphical user interface (GUI).

**Figure 7:**
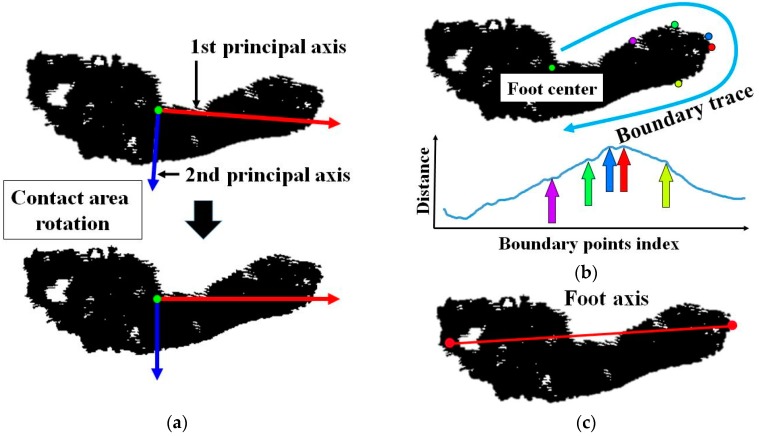
Key point detection process: (**a**) contact region alignment using PCA, (**b**) center of heel detection using boundary tracing, (**c**) definition of the foot axis as a line between the central points of the heel and second toe.

The center of the heel (point K in Figure 1a) is automatically defined using the boundary-tracing technique. In [38], Chun et al. proposed a 3D human pose representation method based on the salient points in the silhouette boundary. To detect the salient points, they first calculated the distances from the centroid of the silhouette to the boundary points by boundary tracing clockwise or counter-clockwise and applied a smoothing filter to the sequentially detected distances. In the sequential distances, the system finds and defines the local maxima as the salient points. Our system performs boundary tracing on the heel side image of the foot center and detects the salient points. Before this process, to recognize the directions to the toe side and heel side, the system aligns the contact region image based on the principle axis computed by PCA, as shown in Figure 7a. The system splits the contact region into two parts based on the centroid. For both parts, the system calculates the maximum width of the contact region perpendicular to the first principal axis. The part with a greater maximum width is defined as the toe side part, and the other is defined as the heel side part. Finally, among the salient points detected in the heel side part, the point farthest from the centroid is selected as the center of the heel (red point in Figure 7b). The line connecting the centers of the second toe and heel is considered the foot axis (Figure 7c). Algorithm 2 explains the foot axis detection procedures.

**Algorithm 2** Procedures of Foot Axis DetectionInput data: contact region Output data: foot axis connecting the centers Variables
-cn: centroid of the contact region-i: index of the boudnary point in the heel side part-$b_{i}$: *i*-th boudnary point in the heel side part-$l_{i}$: distance between cn and $b_{i}$-${\hat{l}}_{i}$: smoothed distance between cn and $b_{i}$-${l^{\prime }}_{i}$: first derivvatnormal vector of ${\hat{l}}_{i}$-j: index of the salient point-$s_{j}$: *j*-th salient point
Procedures
Aligning the contact region:calculate the centroid of contact region cn and rotate the contact region by using PCADefining the heel side part: 2.1.devide the aligned contact region into two parts.2.2.for each part, find maximum width perpendicular to the first principal axis.2.3.define a part with a larger maximum width as a heel side part.Calculating the distances from cn to the boundary points in the heel side part:for all $b_{i}$,calculate distance $l_{i}$ from cn. Smoothing the distances:for all $l_{i}$,compute the smoothed distance ${\hat{l}}_{i}$ using a 1D Gaussian smoothing filter.Deriving the derivative of the smoothed distances:for all ${\hat{l}}_{i}$,compute ${l^{\prime }}_{i}$ the first derivative of the smoothed distance ${\hat{l}}_{i}$ using the central difference method.Detecting the salient points:for all ${\hat{l}}_{i}$,find the local maximum of which ${l^{\prime }}_{i}$ is a zero-crossing point, and define the local maximum as the salient point $s_{j}$.Detecting the center of heel:for all $s_{j}$,find the farthest point from cn and define it as the center of heel.Defining the foot axis:detect the line connecting the detected center of heel and the center of second toe selected by a user.

**Figure 8:**
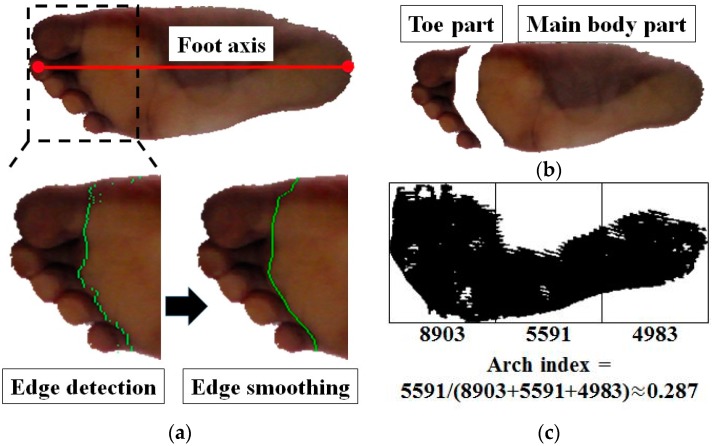
AI computation: (**a**) foot point image rotated by the foot axis (top) and the detected edge (colored green) (bottom), (**b**) toe part and main body part segmented by the edge, (**c**) result of AI computation from the segmented main body part of the contact region.

The AI is the ratio of the area of the middle third of the main body of the contact region to the entire contact region, excluding the toes. The next step is to segment the foot point image into the main body part of the foot and toe area. The edge detection technique is applied for this work, based on the significant color change between the main foot body and toe area due to skin deformation and the shadow, as shown in Figure 6a. To detect the color change, the system first rotates the foot point image based on the foot axis, and divides the image into three parts. The system scans horizontally each line in the toe region, and detects the vertical edges using 3 × 3 Sobel operator (Figure 8a). And in order to find the strongest edge per each scan line, the system performs the non-maximal suppression. The system then applies the smoothing filter to the positions of detected edges by averaging the positions of their neighboring edges. Using these edges, the main part and toe area are divided (Figure 8b). And by applying AND operation to the segmented main part and the contact region, the system detects the main body part of the contact region.

Finally, the system computes the AI by dividing the detected contact region into three parts and calculating the ratio of the area of the middle part to the entire region. Figure 8c shows the example of the AI computation.

#### 4.4.2. Arch Width and Height Computation

The AW and AH are defined as the lengths of the lines from the mid-point of the MBL to the contact region and to the foot point in a direction perpendicular to the footplate. Therefore, the MBL must be measured first. The AW and AH are computed after detecting the mid-point of the MBL.

**Figure 9:**
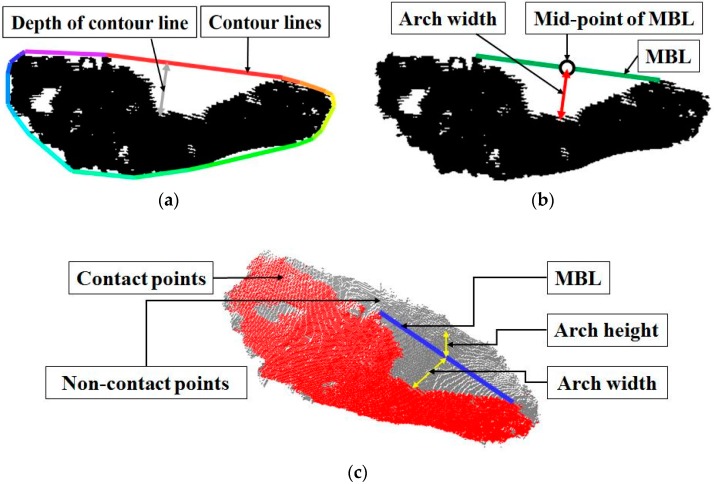
AW and AH computation: (**a**) result of convex hull detection algorithm applied to the contact region image, (**b**) the medial border line (MBL) and the mid-point of the MBL detection and AW computation, (**c**) 3D visualization of the AW and AH computation.

To find the MBL, the system exploits the convex hull detection algorithm [39]. This algorithm finds the convex hull of a 2D point set. The convex hull is the smallest set that contains the entire 2D point set. Through this algorithm, the system obtains the set of contour lines that contains the points in the contact region image (Figure 9a). The line with the greatest depth among the contour lines is defined as the MBL (Figure 9b). The mid-point of the MBL is computed by averaging the two 3D points; i.e., the beginning and end points of the line.

The AW and AH are calculated by defining the two lines perpendicular to the MBL at the mid-point of the MBL. The line for the AW estimation is on the plane (the estimated footplate), begins at the mid-point of the MBL, and intersects with the contact region. The system draws a line perpendicular to the MBL on the contact region image and defines the distance between the mid-point of the MBL to the point on the line intersecting with the contact region as the AW (Figure 9b).

The line for the AH begins at the mid-point of the MBL and intersects with the foot point in a direction perpendicular to the footplate. The system first estimates the line equation using the mid-point of the MBL and the normal vector of the footplate. Using the line equation, the system finds the foot point closest to the line, and defines the distance between the closest foot point and the mid-point of the MBL as the AH (Figure 9c).

## 5. Experiments

In this section, we present experimental results to show that the proposed system facilitates accurate and stable foot-arch parameter measurement in repeat trials. In Section 5.2, we show the accuracy and repeatability of the foot-arch parameters estimated by the proposed system. And in Section 5.3, we show the reliability of the proposed method compared with the ground truth and analyze statistically the measured data. In Section 5.4, we discuss the weight choice for our MRF model used in the contact region detection module. In Section 5.5, the processing time of each module is described. Before reporting the experimental results, we first explain the experimental setting.

### 5.1. Experiment Environment

The experiments to test the feasibility of the proposed system were performed on a 2.83 GHz CPU with 4 Gbyte of memory running 64-bit Windows 8.1 Enterprise K. The Intel Realsense Development Kit was used to capture the color and depth images. OpenCV library was used for image processing, and the Matlab wrapper for graph cut was used to infer the result of MRF-based labeling [40]. Eleven adult volunteers, nine males and two females, aged 25~35 years, participated in the validation. All participants were healthy, with no history of surgery or abnormality that could affect their stand pose. The subjects were asked to stand on the scanning stage and put their right foot on the acrylic board. For each subject, we collected three sets of depth and color images at three different times during a single day. We asked them to return to repeat the above scanning procedure for 3 consecutive days, thus a total of 99 image datasets were obtained.

**Figure 10:**
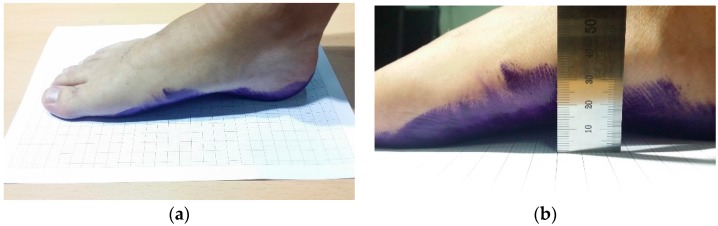
Measuring ground truth data: (**a**) generation of a footprint for AI and AW measurement, (**b**) AH measurement.

To evaluate the accuracy and repeatability of our system, we generated ground truth data for comparison. All the measurements for the ground truth data were performed by a clinical professional, who has more than four years of experience. We first painted the subject’s right foot with ink. Then, the subject placed his/her painted right foot on graph paper (Figure 10a). Using this footprint image, we measured the AI by counting manually the number of painted squares on the footprint image and the AW using a ruler. The AH data were collected by measuring the height from the floor to the skin externally covering the metatarsal bones using a ruler or a caliper, as shown in Figure 10b. We have collected 11 sets of ground truth data on the foot-arch parameters from 11 subjects. For the complete ground truth set, the AI was 23.59~31.14%, the AW was 28~41 mm and the AH was 8~16 mm.

### 5.2. Accuracy and Repeatability

The accuracy was evaluated by comparing the similarity of the measured foot-arch parameters to the ground truth, and the repeatability was determined according to the similarity of the data taken at different time points over 3 days.

Table 1 shows the results of the accuracy and repeatability tests. We performed the foot-arch parameter computation on 99 datasets collected from 11 subjects over 3 days, and compared with the ground truth by calculating the average error (AE). For the repeatability test, we computed the standard deviation (STD) of nine datasets for each subject. For AI computation, average of AE and STD were −0.17% and 0.70%. For AW and AH, averages of AEs were 0.95 mm and 0.52 mm and the averages of STDs were 1.63 mm and 0.68 mm, respectively. The coefficients of variation (CV, mean ÷ standard deviation) that is widely used to express the precision and repeatability are 0.023% for AI, 0.046% for AW, and 0.061% for AH respectively.

### 5.3. Reliability and Statistical Analysis

In this section, we investigate statistically the results of the proposed system to evaluate the reliability and feasibility. To compare the reliability of the estimated foot-arch parameters, correlation coefficients were calculated between the AI, AW, AH and the ground truth. And Spearman’s rank order correlation was also performed between the estimated results and the ground truth. The relationships among the foot-arch parameters, which are still controversial, were also tested.

The correlation coefficients and Spearman’s rank order correlations between the AI, AW, AH estimated by the proposed system and the ground truth were calculated to evaluate the reliability of the proposed method [41]. The correlation coefficients of AI, AW, and AH are 0.798, 0.851, and 0.811 respectively. All the p-value are less than 0.0001. These results show that the foot-arch parameters are significantly correlated with the ground truth. In case of the Spearman’s rank order correlations, the average AI, AW, AH of each subjects measured through the proposed system were used to rank. The result correlation coefficients of AI, AW, and AH are 0.945, 0.923, and 0.95 respectively. All the p-value are less than 0.0001. These results suggest that there are strong positive relationships between the foot-arch parameter measurements collected using the conventional method and the proposed method. Table 2, Table 3 and Table 4 list the individual subject rankings of AI, AW, AH for the ground truth and the proposed method. In case of AI and AH, all of the absolute differences between the two ranks are less than or equal to 2. In case of AW, the absolute difference between two ranks is less than 2 in 10 out of 11 subjects.

**Figure 11:**
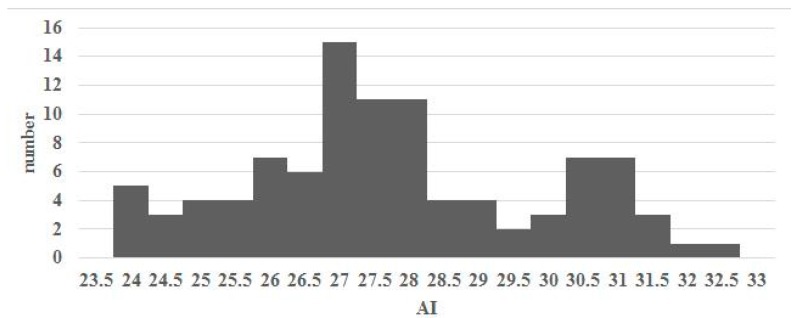
AI distribution for the 99 test data.

Figure 11 shows the distribution of AI measured by the proposed system using 99 data. Cavanagh and Rogers proposed criteria for classifying foot type as high, normal and flat arches using AI calculated from the footprint [4]. Their method involves dividing the distribution of AI into quartiles. The first and third quartiles act as the boundaries to recognize the foot type. For our system, the first and third quartiles were 26.125 and 29.4375. Based on [4], these values suggest that a foot can be recognized as a high arch foot if its AI < 26.125; if the AI > 29.4375, then a foot can be recognized as a low arch foot. If its AI falls between these values, it can be recognized as a normal arch foot.

**Figure 12:**
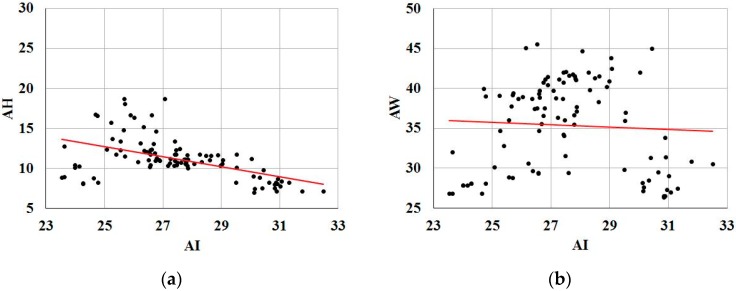
Linear regression analysis for 99 datasets: (**a**) scatter plot showing regression between AI and AH (correlation coefficient r = −0.51, p < 0.0001), and (**b**) scatter plot showing regression between AI and AW (correlation coefficient r = −0.06, p = 0.71).

Figure 12 shows a linear regression analysis of the relationships among AI, AH and AW for 99 datasets measured by the proposed system. The correlation coefficient between AI and AH was a negative value (*r* = −0.51) and was statistically significant (*p* < 0.0001). AI and AW were less strongly correlated (*r* = −0.06), and the result was not significant (*p* = 0.71).

### 5.4. Weight for the MRF Model

In the contact region detection, the system extracts the dense and connected point set to be the estimated region in contact with the footplate by minimizing iteratively the energy function in the proposed MRF model. The energy function consists of four terms-color, distance, angle, and smoothness potentials—and it has weights, $w=\left\{w_{\psi },w_{\lambda },w_{\rho }\right\}$, for the first three terms as the control values that enable regulation of the relative importance of each term. In this section, we investigate the changes in foot-arch parameters according to the changes in weights, and propose the optimal weights.

We search for an automatic criterion to find the optimal weights for the accurate estimation of foot-arch parameters. We first define a cost function relating the errors of estimated foot-arch parameters to changes in the weights:

(6) $$F\left(w\right)=\frac{1}{2}\sum_{i=1}^{m}{\left(f_{i}\left(w\right)\right)}^{2}=\frac{1}{2}{\left\|f\left(w\right)\right\|}^{2}=\frac{1}{2}f{\left(w\right)}^{T}f\left(w\right),\;f_{i}\left(w\right)=G_{i}-E_{i}\left(w\right)$$

where m is the number of samples, $G_{i}$ and $E_{i}\left(w\right)$ are the i-th ground truth and estimated result, and f is a vector function: $f:\;{\mathbb{R}}^{n}\to {\mathbb{R}}^{m}\;$ with m≥n, n is the dimension of w. Here, we want to find the optimal weights $w^{*}$ that minimize F(w) and equivalently minimize ‖f(w)‖. To find the minimizer $w^{*}$, we apply the Levenberg-Marquardt algorithm (LMA) [42]. We start with an initial guess, $w_{0}$. In the iterations, w is updated by $w_{\mathrm{new}}=w+h$ only for the downhill step. The step h is calculated by solving $\left(J{\left(w\right)}^{T}J\left(w\right)+\mu I\right)h=-J{\left(w\right)}^{T}f\left(w\right)$, where $J\left(w\right)\in {\mathbb{R}}^{m\times n}$ is the Jacobian of derivatives of f(w) with respect to the weights and μ is the adaptive damping parameter. If $\frac{1}{2}{\left\|f\left(w_{\mathrm{new}}\right)\right\|}^{2}>\frac{1}{2}{\left\|f\left(w\right)\right\|}^{2}$, then reject the step, keep the old w  and the old f(w), adjust damping parameter μ, and calculate the step h again. If f(w) is converged during the iterations, return w as the minimizer of the cost function  F(w). The proposed system outputs three different estimation results for the AI, AW, and AH. Therefore, there are three minimizers for each foot-arch parameter. To calculate them, we applied the above LMA-based method for AI, AW, and AH separately. Among the 99 data, 22 were chosen randomly and used for the sample (m = 22), and the remaining data were used for the evaluation. The calculated optimal minimizers and the average error of the 77 evaluation data are shown in Table 5.

### 5.5. Processing Time

We tested the processing time using 99 dataset, and calculated the average time for each submodule of analysis module. As shown in Table 6, the processing times of three submodules, with the exception of the contact region detection, were less than 60 ms. The processing time of the contact region detection was 8301.62 ms, which is the most time consuming.

## 6. Discussion

In this paper, we developed autonomous foot-arch parameter measurement system for estimating three foot-arch parameters, AI, AW, and AH, through the use of a RGB-D camera. The system makes use of well-known image processing techniques, such as normal vector clustering, MRF-based segmentation, and 2/3D morphology and shape analysis, to detect the contact region and key features of foot and to calculate the foot-arch parameters.

In Section 5.2, we described the accuracy and repeatability of the foot-arch parameters obtained by the proposed system. And the mean error rates (=|AVG AE|/AVG GT×100) of AI, AW, and AH were about 0.6%, 2.7%, and 4.6%, respectively. This may result not only from computational errors in the processing of the data but also from inherent measurement noise of the RGB-D camera. In fact, the RGB-D used in the proposed system is known for having an around 0.5~0.6% detecting error on the distance [21]. Therefore, it is expected to obtain more accurate and sophisticated foot-arch parameters if the performance of the RGB-D camera has been improved.

As far as we know, lots of existing methods estimate the parameters related with the foot or arch, but there is no system that calculates the AI, AW, and AH simultaneously. Therefore it is difficult to directly compare the accuracy and repeatability of the proposed method with the previous studies. The method proposed in [17] calculates the ball width, ball girth, instep height, and instep girth using several 12 RGB cameras. The average estimation error of their method is about 2 mm. The method proposed in [29] estimates the foot length, width and ball girth using a RGB-D camera. The standard deviations of measurements of 10 times for each subject’s foot length and width are both 3.5 mm. In the case of the ball girth, the variations are about 6 mm. The digital image processing based AI computation study proposed in [30] shows that the coefficient of variation (mean ÷ standard deviation) of AI measurements of 10 times is 1.16%. As shown in Table 1, although the comparison parameters and evaluation dataset are different, the accuracy and repeatability of the proposed system for AI, AW, and AH measurements are relatively better.

In Section 5.3, we firstly tested the reliability of the proposed system and showed that the foot-arch parameters estimated through the proposed method are strongly correlated with the ground truth measured by the conventional method. Also the ranks of foot-arch parameters using both methods are similar. This means that the foot-arch parameters measured by the proposed method can be used to identify the feature of individual foot as the foot-arch parameters measured by the conventional method do. However, in case of AI, the correlation coefficient is relatively smaller than the others. In the procedure of AI calculation, the system extracts the edge between main body part of the foot and toe area, and then calculates the ratio of middle area of main body over total main body. The edge is detected based on the color difference between the main body and toe area, and the system defines the edge if the color difference between the neighboring pixels is greater than a predefined threshold (in the proposed system, we defined the threshold as 50 explicitly.). Thus, the stable lighting condition is important to solve this problem which is a common and significant issue in computer vision application systems. In order to alleviate the problem caused by the different lighting condition, we installed a LED desk lamp inside of the scanning stage. We located the lamp in front of the toe direction to emphasize the color difference between the main body and toe area.

Many studies reported the correlations among the foot-arch parameters have come to the controversial conclusion, particularly that between the AI and AH [4,30,43,44,45]. Despite these controversy, the correlation coefficient between the AI and AH generally demonstrated similar results showing negative correlation (e.g., −0.70 in [30], −0.67 in [43], −0.39 in [44], and −0.42 in [45]). As shown in Figure 12, the correlation between AI and AH obtained from our system is consistent with these previous studies. On the other hand, according to the results of the correlation between AI and AW, these two parameters are not related to each other. These results indicate that the analysis result based on the data extracted from the proposed system is not different from the results of existing researches, and show the availability of the proposed system to biomedical researches for foot analysis.

In Section 5.4, we introduced a method to determine the optimal weight of each term to find the contact region by using the MRF method. We found that the optimal weights are different depending on the foot-arch parameter. Nevertheless, it was confirmed that the difference in arch parameters was calculated using different optimal weights. As shown in Table 5, the weight $w_{\lambda }$ for the second term, distance potentials, is higher than the others in three cases, although their values are different. These results indicate that distance potentials are significantly considered for accurate foot-arch parameter estimation. This is also in accord with the definition of the contact region: the distance from the plane of the contact points is close to 0. The average errors of the estimation results using three different weight sets were less than 0.57%, 1.03 mm, and 0.74 mm, and the differences were less than 0.74%, 0.08 mm, and 0.22 mm, respectively.

In Section 5.5, we presented the processing time of each submodule in the analysis module. The proposed system outputs the foot-arch parameters on average within 8.5 s. Compared with the system proposed in [30] that takes 10~30 s for AI computation from a footprint image, the proposed method is relatively fast. Among the submodules, the contact region detection consumes more than 98% of the total processing time. This module solves the contact region detection problem by iteratively minimizing the energy function and finding the optimal label set for each pixel. To reduce the processing time of this module, GPU-based parallel processing can be applied. According to [46], a GPU-based solver for pixel labeling problems is 10–12-fold faster than the CPU-based solver used in this study. In the case of the proposed system, we did not apply a parallel-processing technique, since the system dealt with a single depth and color image set for a static pose. However, if the number of data processed by the system increases, the processing time of this module could be a problem. We are considering a parallel-processing technique to improve the applicability of the system as future works.

From the automation perspective of foot-arch parameter computation, the proposed system automatically performs the rest of the processes except for the foot axis definition for AI calculations. In order to define the foot axis, the proposed system specifies the center point of the second toe through the graphical user interface. For this purpose, a color image of the foot obtained is displayed to the monitor, and the user sets the center point by clicking the mouse manually. In addition, it provides the function to correct the designated point so that accurate foot axis detection is possible. Although this manually selection does not require much time (less than 2 s on average in the experiment on 99 data), the usability of the proposed system will be more improved if this method becomes also automated. However, as opposed to automatically detected the center point of heel as the salient point on the boundary in the foot-heel, it is difficult to detect the center point of the second toe by image processing or computer vision techniques due to the lack of visual, geometrical, and topological features. In this case, machine learning-based detection method can be a good solution [8]. We will apply the automatic detection of the center point of the second toe to the proposed system. We will also apply the machine learning to estimate the position and area of important anatomic structures of foot, such as the calcaneus, the talus, the navicular bone, and the metatarsal bones, which are known only through X-ray, CT or MRI, from color and depth images taken by the proposed system.

One of the most difficult and important parts in the proposed system is the contact region detection. Most existing foot-arch parameters such as AI, AW, AH, arch length index, footprint index, arch angle, truncated arch index, and so on, are based on the shape of contact region [21]. This is since the individual musculoskeletal structures of the feet are reflected onto the contact region. Therefore, the detection of the contact region is very important and can be applied to lots of foot-related researches. The proposed system detects the contact region using the MRF as described in Section 4.3.2. Another possible method to detect the contact region is Active shape models (ASMs) [47]. The ASMs, widely used in facial image analysis and medical imaging, is a statistical model that iteratively deforms the shape of the given model to fit it to the desired shape of an object. Assuming that each person's contact region is not significantly different, the ASMs can be applied to contact region detection. In particular, this will allow the system to detect the contact region faster than used MRF based method, since the processing time of ASMs is very fast.

One of the representative advantages of the proposed system is that the system is able to capture the full 3D geometric plantar shape of the foot sequentially. Unlike foot pressure-based method which measures the pressure due to the body weight applied to the foot contact region, the proposed system enables to obtain 3D information of the plantar shape of the foot including the contact region. Also, unlike the existing 3D foot shape acquisition method, the proposed method can be used for data measurement and analysis of 3D foot shape change according to continuous and dymamic motions, such as gait, running, squat, and jumping, since continuous data acquisition is possible. Especially, although the most of existing studies investigating the change of the arch according to the posture change only consider the static postural changes such as sitting and standing, it is possible to efficiently observe the continuous shape change of the foot-arch according to various motions using the proposed system.

## 7. Conclusions

In this paper, we presented a RGB-D camera-based geometric foot-arch measurement system that is able to capture the sole of the foot and estimate three foot-arch parameters: AI, AW and AH. To achieve these goals, the proposed system provides the following: (1) 3D measurement of the plantar surface of the foot, (2) detection of the contact region, and (3) AI, AW, and AH estimation via 2/3D shape analysis of the contact region.

The feasibility of the system was proven by the four tests which are the average estimation error measurements, statistical analysis, optimal weights used in the MRF, and the processing time. From the tests it was validated that the proposed system can be used to obtain reliable geometric information of the foot plantar surface and foot-arch parameters.

Our future work will focus on expanding the applicability of our system to dynamic foot measurement and recognition, such as a gait and run analysis. More sophisticated methods that take into account automatic foot part tracking and recognition will improve the feasibility and suitability of the proposed system for dynamic foot analysis. As stated in the discussion, the parallel-processing technique for the contact region detection module and the machine learning for automatic foot region segmentation and landmark detection will be considered to reduce the processing time and increase the efficiency of the system. The use of our system to identify novel characteristics of static and dynamic foot analyses, such as the relationship between the foot-arch parameters and personal gait patterns, is an important future research topic.

## Figures and Tables

**Table 1:** Accuracy and repeatability assessment.

Subject	AI (%)			AW (mm)			AH (mm)		
	GT	AE	STD	GT	AE	STD	GT	AE	STD
1	29.06	1.63	0.89	29	1.86	1.77	8	−1.06	0.51
2	31.14	−0.40	0.37	28	−0.86	2.49	8	0.95	0.63
3	25.27	−1.35	1.12	30	0.67	0.99	11	1.93	0.87
4	27.90	−0.81	0.96	37	1.71	1.93	12	2.06	0.56
5	23.59	−1.29	0.28	28	−0.65	0.57	9	0.57	0.84
6	26.66	0.95	0.36	40	1.38	2.90	12	0.49	0.56
7	28.26	0.60	0.48	41	1.12	1.21	11	0.38	0.45
8	25.86	−1.34	0.85	35	1.76	1.97	14	−1.00	1.15
9	26.73	−0.02	0.79	38	2.24	0.41	16	1.29	1.07
10	29.30	−0.21	1.23	41	−0.80	1.93	10	0.42	0.43
11	27.75	0.33	0.38	39	2.05	1.74	11	−0.31	0.44
AVG	27.41	−0.17	0.70	35.09	0.95	1.63	11.09	0.52	0.68
CV(%)	0.023			0.046			0.061		

GT: Ground Truth, AE: Average Error, STD: Standard Deviation, AVG: Average, CV: Coefficient of Variation.

**Table 2:** Individual subject rankings based on AI.

Subject	Ground Truth		Proposed Method		Rank Difference
	AI	Rank	AI	Rank	
5	23.59	1	22.3	1	0
3	25.27	2	23.92	2	0
8	25.86	3	24.52	3	0
6	26.66	4	27.61	6	−2
9	26.73	5	26.71	4	1
11	27.75	6	28.08	7	−1
4	27.9	7	27.09	5	2
7	28.26	8	28.86	8	0
1	29.06	9	30.69	10	−1
10	29.3	10	29.09	9	1
2	31.14	11	30.74	11	0

**Table 3:** Individual subject rankings based on AW.

Subject	Ground Truth		Proposed Method		Rank Difference
	AW	Rank	AW	Rank	
2	28	1.5	27.14	1	0.5
5	28	1.5	27.35	2	−0.5
1	29	3	30.86	4	−1
3	30	4	30.67	3	1
8	35	5	36.76	5	0
4	37	6	38.71	7.5	−1.5
9	38	7	40.24	8	−1
11	39	8	41.05	9	−1
6	40	9	41.38	10	−1
7	41	10.5	42.12	11	−0.5
10	41	10.5	40.2	7.5	3

**Table 4:** Individual subject rankings based on AH.

Subject	Ground Truth		Proposed Method		Rank Difference
	AH	Rank	AH	Rank	
1	8	1.5	6.94	1	0.5
2	8	1.5	8.95	2	−0.5
5	9	3	9.57	3	0
10	10	4	10.42	4	0
3	11	6	12.93	8	−2
7	11	6	11.38	6	0
11	11	6	10.69	5	1
4	12	8.5	14.06	10	−1.5
6	12	8.5	12.49	7	1.5
8	14	10	13	9	1
9	16	11	17.29	11	0

**Table 5:** Assessment optimal minimizer and error of foot-arch parameter estimation of the 77 data.

	AI	AW	AH
minimizer $w^{*}=\left\{w_{\psi }^{*},w_{\lambda }^{*},w_{\rho }^{*}\right\}$	{2.9, 9.3, 1.8}	{4.7, 6.7, 0.9}	{6.2, 7.3, 1.2}
average AE {AI(%), AW(mm), AH(mm)}	{−0.17, 0.95, 0.52}	{0.57, 1.03, 0.74}	{0.54, 0.99, 0.62}

**Table 6:** Processing time of the analysis module.

Module	Task	Processing Time (ms)
Preprocessing	Noise removal/coordinate mapping	53.51
Contact region detection	Contact point detection	51.4
	MRF-based region segmentation	8301.62
Foot-arch parameter computation	AI, AW, AH estimation	39.97
Total	-	8446.5
